# Cross-modal commutativity of magnitude productions of loudness and brightness

**DOI:** 10.3758/s13414-021-02324-y

**Published:** 2021-06-08

**Authors:** Wolfgang Ellermeier, Florian Kattner, Anika Raum

**Affiliations:** Institute of Psychology, Technical University of Darmstadt, 64283 Darmstadt, Germany

**Keywords:** Magnitude estimation, Cross-modality matching, Cross-modal commutativity, Axiomatic measurement, Psychophysics

## Abstract

In their fundamental paper, Luce, Steingrimsson, and Narens (2010, *Psychological Review, 117*, 1247-1258) proposed that ratio productions constituting a generalization of cross-modality matching may be represented on a single scale of subjective intensity, if they meet “cross-dimensional commutativity.” The present experiment is the first to test this axiom by making truly cross-modal adjustments of the type: “Make the sound three times as loud as the light appears bright!” Twenty participants repeatedly adjusted the level of a burst of noise to result in the desired sensation ratio (e.g., to be three times as intense) compared to the brightness emanating from a grayscale square, and vice versa. Cross-modal commutativity was tested by comparing a set of successive ×2×3 productions with a set of ×3×2 productions. When this property was individually evaluated for each of 20 participants and for two possible directions, i.e., starting out with a noise burst or a luminous patch, only seven of the 40 tests indicated a statistically significant violation of cross-modal commutativity. Cross-modal monotonicity, i.e. checking whether ×1, ×2, and ×3 adjustments are strictly ordered, was evaluated on the same data set and found to hold. Multiplicativity, by contrast, i.e., comparing the outcome of a ×1×6 adjustment with ×2×3 sequences, irrespective of order, was violated in 17 of 40 tests, or at least once for all but six participants. This suggests that both loudness and brightness sensations may be measured on a common ratio scale of subjective intensity, but cautions against interpreting the numbers involved at face value.

## Introduction

Cross-modality matching assumes a pivotal role in Stevens’ (1975) “New Psychophysics.” By matching sensations on one modality, for example, loudness, to equal sensation magnitudes on another modality, for example, brightness, the resulting cross-modality matching function provides an empirical consistency check for the independently determined unimodal psychophysical power functions obtained via magnitude estimation. In theory, the power function exponent of the matching function should simply be the ratio of the two unimodal power functions; which, in the relatively few direct empirical tests of this proposition, has been found (e.g., loudness and line length: Collins & Gescheider, 1989; Hellman & Meiselman, 1990; brightness and loudness: Stevens & Marks, 1965; Walsh & Browman, 1978: pain and handgrip strength: Gracely et al., 1978). It is further stipulated that if cross-modality matches pass this test, a common underlying scale of sensory magnitude – independent of modality – may be assumed. Of course, this reasoning relies on what has been pejoratively labelled a “curve-fitting” approach, as opposed to a mathematically formulated psychophysical theory.

## Axiomatic theory of magnitude scaling

The first formal conceptualizations of cross-modality matching (Krantz, 1972; Krantz et al., 1971) contrasted a “mapping theory” of cross-modality matching with the eventually adapted “relation theory” (Shepard, 1981) emphasizing the role of sensation ratios rather than the subjective magnitude of single stimuli. However, it was not until Louis Narens’ (1996) seminal paper that a comprehensive theory of magnitude scaling, not just cross-modality matching, based on axiomatic measurement theory emerged. His thoroughly formulated theory basically states that in order for magnitude estimates (as well as magnitude productions, or cross-modality matches) to be valid, two empirically testable axioms have to hold: (1) commutativity (Narens’ Axiom 4) and (2) multiplicativity (Narens’ Axiom 9).

Commutativity means (in a simplified notation compared to Narens, 1996) that given *x* is a stimulus intensity produced in a magnitude production trial, and *p* and *q* are positive numbers, adjusting a physical stimulus to have *p* times the subjective magnitude as some reference intensity (thereby resulting in *x*_p_), and subsequently, starting from that outcome, to produce another stimulus intensity *q* times as strong subjectively (i.e., *x*_p,q_) should result in the same stimulus intensity as performing the two operations in the reverse order, i.e.:

1 $$ {x}_{p,q}\sim \kern0.5em {x}_{q,p} $$

For the case of magnitude productions of loudness that means that first doubling the loudness of a given reference sound pressure level, and on some subsequent trial tripling the outcome of that first trial, should result in the same sound pressure level as initially tripling and then doubling loudness starting from the same reference level.

Multiplicativity means that successive ratio productions actually result in the implied numerical outcome: Thus, making a reference level *p* times as intense, and the outcome *q* times as intense in turn, should produce the same stimulus level as making the reference ***r*** times as intense in a single adjustment, with *r* = *pq*:

2 $$ {x}_{p,q}\sim \kern0.5em {x}_r $$

In our example, a sequence of first making a reference sound twice as loud and subsequently producing a level that is three times as loud as the outcome should result in the same sound pressure level as adjusting a level six times as loud as the reference in a single shot.

Narens (1996) has shown that, if commutativity – together with a number of technical axioms – holds, then subjects are operating on a ratio scale of sensation. If both commutativity and multiplicativity hold, then the numerical ratios instructed in the magnitude production task may be taken as veridical, i.e. as true mathematical numbers (which may be interpreted like in algebraic multiplication).

## Cross-modal commutativity in magnitude production

Luce et al. (2010) extended Narens’ (1996) theory of magnitude estimation/production and Luce’s (2004) “global psychophysical theory” to apply to cross-modal magnitude production. The latter refers to a task in which a participant is asked to, for example, “Make a light three times as bright as the sound is loud!”, an extension of both cross-modality matching (the case of producing equal sensation magnitude across modalities) and magnitude production (producing multiples of sensations on a single dimension). While in their theoretical account, Luce et al. (2010) distinguish a great number of cases (unimodal, mixed-modality, fractionation, and ratio production) for this “generalized” commutativity to be tested, the crucial case is stated in their proposition 3 as involving a mapping from one dimension into another and back:

3 $$ {x}_{p,q}^{fgf}\sim \kern0.5em {x}_{q,p}^{fgf} $$

where the subscripts *q*, *p* refer to magnitude production factors as before, and the superscripts *f*, *g* represent the dimensions/modalities involved, with the single cross-modal production trial (*f* → *g*) spelled out above (in words) being formalized as ${x}_p^{fg}$ and *f* representing sound pressure level, *g* representing luminance, and *p* being the production factor, here *p* = 3. Consequently, ${x}_{p,q}^{fgf}$might be a mapping from loudness to brightness and back to loudness (*f* → *g* → *f*), with *x* (on both sides of Eq. 3) being a sound pressure level. Likewise, an analogous case exists where the two successive operations originate from the brightness continuum (*g* → *f* → *g*), and consequently *x* refers to a luminance level:

4 $$ {x}_{p,q}^{gfg}\sim \kern0.5em {x}_{q,p}^{gfg} $$

Thus, cross-modal commutativity as specified by Luce et al. (2010) offers two independent and – due to the different physical continua they are measured on – not directly comparable opportunities to test the validity of this property, depending from which modality the cross-modal productions originate.

## Earlier tests of cross-dimensional commutativity

In addition to formulating a theory of cross-modal psychophysics, Luce et al. (2010) also tested selected cases of their theory, though on a relatively small number of participants each. The experimental paradigm they employed addresses what they call “cross-dimensional commutativity” in that loudness of 1-kHz tones is mapped into the loudness of 2-kHz tones, thereby involving two different physical dimensions *f* and *g*, but no change between sense modalities; the sensation with respect to which the judgments/productions are made is always loudness. As to the critical case of “cross-dimensional commutativity,” they found all four participants studied in that condition (two using multiples 2 and 3, and two using proportions 50% and 75%; see their Table B.2) to exhibit cross-dimensional commutativity (re Eq. 3). However, the cross-modal productions *f* → *g* → *f* did not coincide with unimodal productions *f* → *f* → *f*. Luce et al. (2010) interpret this result as indicating that sensation magnitudes on the two dimensions are measured on a common scale, but with different reference points when mapping a sensation from *f* into *g* and vice versa, an issue that is taken up again in the *Discussion* section.

When Steingrimsson et al. (2012) replicated these “cross-dimensional” tests using luminous patches of different hues, the four participants for whom productions of the type *f* → *g* → *f* were evaluated all exhibited cross-modal commutativity (as may be read from their Fig. 7), but the net result of these productions again did not agree with the unimodal case.

## Goals of the present study

It is interesting to note that none of the empirical tests of the sophisticated axiomatic theory of “global psychophysics” (Luce et al., 2010) included truly cross-modal matches or productions. The two test cases published by the authors along with their theoretical work – loudness across different frequencies (Luce et al., 2010) and brightness across different hues (Steingrimsson et al., 2012) – are what they call “cross-dimensional” paradigms in that the attribute to be adjusted (e.g., loudness) remains the same, even though the physical dimension that is manipulated (tone frequency) might vary. Towards the end of their programmatic article, Luce et al. (2010) envision: “… the next step is to extend the model to intermodal situations” (p. 7). That is the focus of the present work in that mappings between two different sensory modalities (hearing and vision) are investigated, and – consequently – two different psychophysical attributes are judged (loudness and brightness), thereby studying actual cross-modal magnitude production.

The focus is on evaluating *cross-modal commutativity* (as in Eqs. 3 and 4), but two other conditions resulting from Narens’ (1996) theory are also investigated in a cross-modal paradigm: The *monotonicity* of the adjustments, meaning that for *p* > *q*, a *p*-times production will yield a greater stimulus intensity on the target dimension than a *q*-times production:

5 $$ {x}_p^{fg}\succ {x}_q^{fg} $$

Furthermore, the *multiplicativity* of cross-modal productions is investigated, thereby extending Eq. 2 to the cross-modal situation:

6 $$ {x}_{p,q}^{fgf}\sim \kern0.5em {x}_{1,r}^{fgf} $$

Note, that here, for example, a ×2×3 sequence is compared with a ×1×6 sequence to test multiplicativity, since with a simple cross-modal ×6 adjustment we would not end up on the same dimension.

Conceptually, if monotonicity holds, one may safely assume that participants generate cross-modal matches/productions on an ordinal scale. Commutativity, if valid, implies observers use a common ratio scale in making cross-modal magnitude productions (with some limitations to that conclusion to be discussed), and multiplicativity would justify taking the instructed sensation ratios ( *p*, *q*) at “face value”, i.e. interpreting them as mathematical numbers.

## Method

### Participants

A total of 21 participants, including the first author, completed the experiment. One of them had to be excluded, because she admitted to inconsistently having evaluated the brightness of the square to be judged/adjusted on some trials, and on other trials its darkness. The remaining sample consisted of 12 women and eight men ranging in age between 18 and 62 years (*MD* = 24). Most of the participants were students of psychology or cognitive science who took part for course credit. All reported normal hearing and normal or corrected-to-normal vision. The protocol of the present research was submitted to the central ethics commission of the Technical University of Darmstadt and found to be uncritical (EK 24/2019).

### Apparatus and stimuli

The experiment was conducted in a double-walled, sound-attenuated chamber (iac acoustics, Niederkrüchten, Germany) in the basement of the department building. Stimulus presentation and response registration were programmed in MATLAB utilizing the Psychophysics Toolbox extensions (Brainard, 1997; Pelli, 1997).

The sounds to be adjusted were digitally generated 500-ms bursts of pink noise with rise/decay times of 10 ms. They were D/A converted by an external sound card (RME Multiface II) with 16-bit resolution at a sampling rate of 44.1 kHz, passed through a headphone amplifier (Behringer HA 8000 Powerplay PRO-8), and played back diotically via electrodynamic headphones (Beyerdynamics DT 990 PRO). Sound levels were verified at the headphones using a sound level meter (Brüel & Kjær 2250) and an artificial ear (Brüel & Kjær Type 4153).

The light sources to be adjusted were luminous 5.7 × 5.7 cm grayscale squares presented on a regular TFT monitor (1,280 × 1,024 pixels) on a black background (approx. 0.2 cd/m^2^). The pixel intensity of the square was adjustable between 0 (black) and 255 (white), corresponding to luminance values of 0.2 cd/m^2^ and 85 cd/m^2^. Luminance levels were verified using a photometer (L 1009, Lichtmesstechnik Berlin) and resulted in a very good fit to a power function relating pixel intensity (*P*) to luminance (*L*_*v*_) by

$$ {L}_v=0.003\times {P}^{1.857}-1.076. $$

### Procedure

#### Cross-modal magnitude productions

On each trial, participants were asked to make a cross-modal magnitude production either from brightness to loudness, or vice versa. To that effect, both a noise burst and a grayscale square were presented simultaneously for 500 ms accompanied by an instruction on the screen, for example, “Adjust the loudness of the sound to appear twice as intense as the brightness of the square!” Starting levels for the variable stimuli were randomly selected from the midrange, i.e. between 50 and 65 dB(A) for sounds and between 0.5 and 50 cd/m^2^ for the luminous squares. Participants then adjusted the level of the variable stimulus by using two sets of “buttons” on the screen interface: Clicking the computer mouse on buttons labelled ‘>’ and ‘>>’ increased sound pressure levels by 1 and 6 dB, respectively, and pixel intensities by 3 and 15 units (on a scale from 0 to 255), to provide both small and large step sizes. Buttons labelled ‘<’ and ‘<<’ decreased levels by the same amounts. After participants had clicked one of the buttons, the audiovisual stimulus combination was repeated at the adjusted level, and so forth, until they pressed the “enter” key to indicate the match was satisfactory. When participants hit the limit of the permissible stimulus range, i.e., 90 dB SPL or 85 cd/m^2^, a message “maximal loudness (or brightness) reached” was displayed.

#### Types of trials for testing axioms

**Fig. 1:**
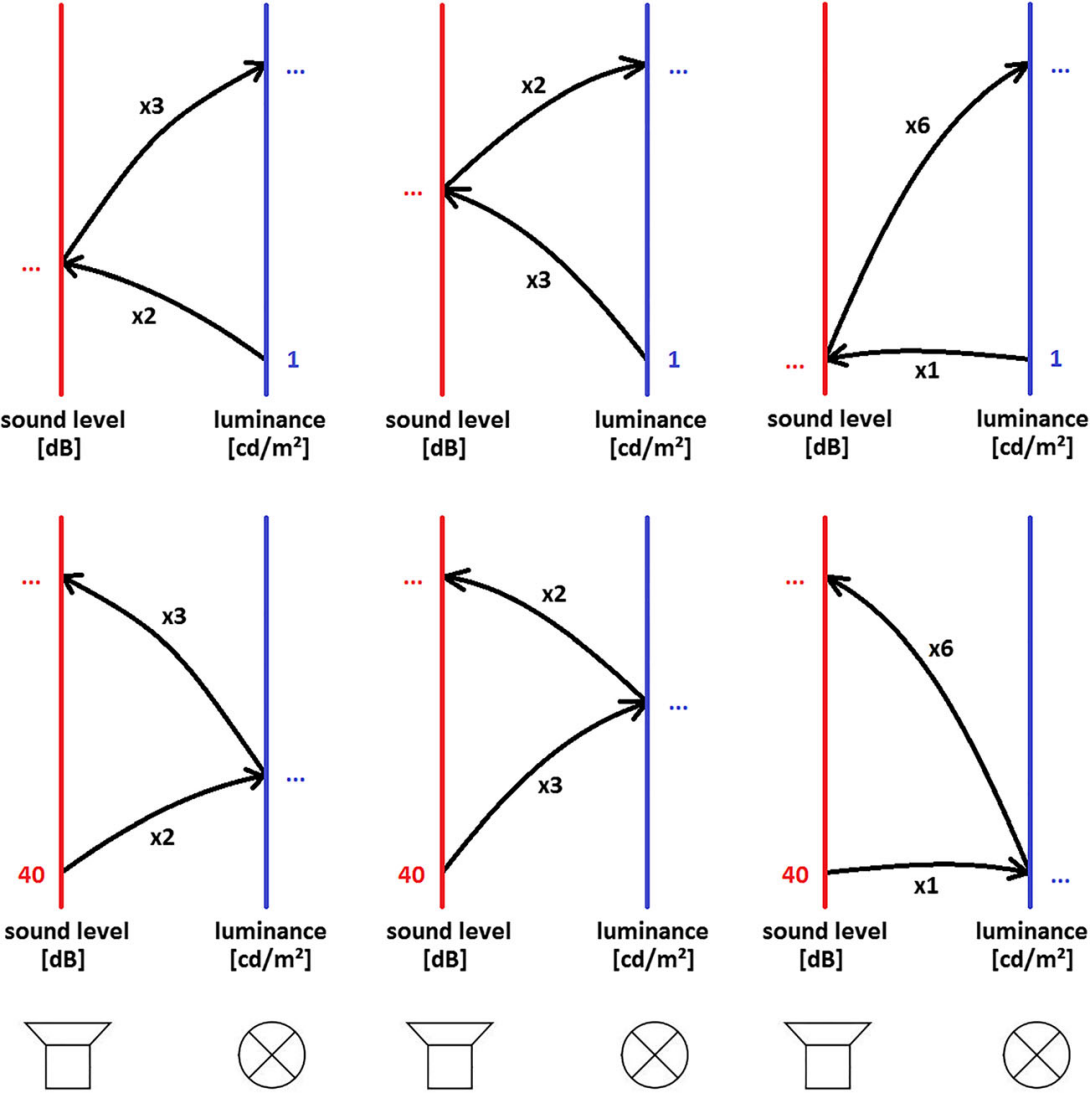
Depiction of the 12 types of cross-modal magnitude production trials. **Top row:** Mappings from brightness (*b*) to loudness (*l*) and back (*x*^*blb*^). **Bottom row:** Mappings from loudness to brightness and back (*x*^*lbl*^). Left column: ×2×3 productions (a doubling of magnitude followed by a tripling); center column: ×3×2 productions; right column: ×1×6 productions (a match followed by a sixfold increase)

Evaluating the axioms of cross-modal commutativity and multiplicativity requires implementing different types of magnitude production trials, which are illustrated in Fig. 1. *Basic* trials are ×2, ×3, and ×1 adjustments originating from standard levels of 40 dB(A) for pink noise and 1.66 cd/m^2^ for the luminance of the square (bottom arrows in each of the six graphs in Fig. 1) and producing the respective sensation magnitude in the other modality. For *successive* trials, a prior adjustment serves as the standard based on which the perceived magnitude on the other modality is adjusted to be ×2, ×3 and ×6 times as intense (top arrows in each of the six panels of Fig. 1). These 12 types of trials (six basic, six successive) were randomly presented in a block of trials, with the obvious constraint that a given successive trial be preceded by the basic trial it builds upon. Note that the final outcomes of all successive trials in the top row of Fig. 1, i.e. three luminance values, should agree, if the axioms of commutativity and multiplicativity hold. The same holds for the three types of cross-modal, successive adjustments depicted in the bottom row of Fig. 1, originating from the loudness continuum and resulting in final sound pressure levels that should coincide.

#### Session structure

Each participant completed the experiment in two sessions lasting approximately 45–60 min. A session consisted of seven blocks of trials – with optional pauses in-between – each of which contained the 12 trial types specified in the previous section, thus resulting in a total of 168 (2 × 7 × 12) cross-modal magnitude production trials per participant.

## Results

### Individual descriptive data

**Fig. 2:**
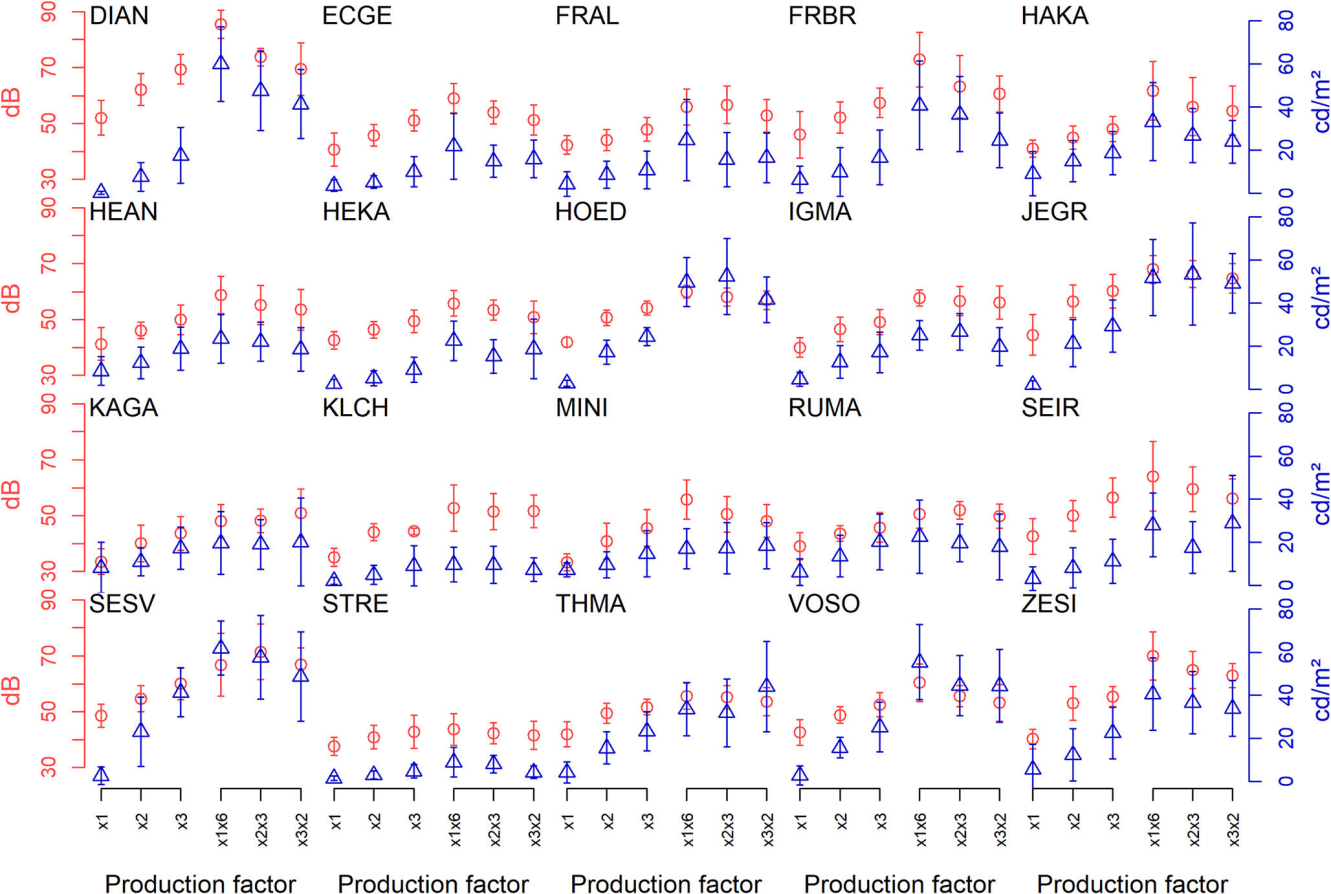
Mean magnitude productions made by all 20 participants. For each participant, the mean adjustment for each of 12 trial types (as illustrated in Fig. 1) is shown along with its standard deviation. On the abscissa, the six types of simple or successive trials are marked, the two ordinates refer to adjusted sound pressure levels (left) or luminance values (right). The adjustments are shown for cross modal productions ending up on the loudness continuum (red circles), and for those (eventually) producing a brightness value (blue triangles). The left part of each graph shows simple cross-modal adjustments, the right part successive adjustments resulting from the concatenation of two cross-modal operations

Each participant’s data were analyzed individually and all axiom testing was performed on within-subjects comparisons. For graphical inspection, each participant’s adjustments were arithmetically averaged across repetitions per trial type. The results are shown in Fig. 2 for each of the 20 participants. It is evident that the adjustments of individual participants cover different stimulus ranges while the dispersion of the 14 repetitions per condition (indicated by the error bars) tends to be of similar magnitude across participants.

Two participants hit the ceiling of the stimulus range at least once in the course of roughly half of their trials (DIAN for sound level; SESV for luminance), but that did not seem to affect the pattern of outcomes across the different conditions (see Fig. 2). Considering each and every level change made in the course of the experiment by all participants, the maximal sound pressure level of 90 dB SPL was reached in only 0.016% of all adjustments (i.e., mouse clicks) and the luminance maximum of 85 cd/m^2^ in a mere 0.17%. Note that exploring the range of attainable stimulus levels may well be part of a reasonable magnitude production strategy.

Descriptively, the basic cross-modal magnitude productions of “making a given loudness equally (×1), twice (×2), and three times (×3) as bright” (or their equivalents with switched modalities) tend to increase monotonically (see the increasing values in the left half of each graph). When inspecting the successive, two-stage cross-modal adjustments displayed in the right half of each graph, it is evident that they do not quite end up at the same stimulus level as they should, if both commutativity and multiplicativity hold. Particularly, the successive adjustment of ×1×6 tends to exceed the outcome of the two ×2×3 (×3×2) adjustments, thus casting doubt on the validity of the multiplicative axiom for most participants.

### Statistical analysis

In order to statistically analyze the validity of each of the relevant measurement axioms, two strategies were employed: (1) Non-parametric null hypothesis testing, and (2) a Bayesian approach. These statistical analyses were performed on the individual sound-pressure level adjustments in decibels and on the grayscale adjustments after converting from the recorded pixel intensity to luminance in candela per m^2^.

Monotonicity was assessed by first performing Friedman analyses of ranks on the ×1, ×2, and ×3 basic adjustments, followed by pairwise Wilcoxon post hoc tests to locate significant differences. Commutativity was evaluated using Wilcoxon signed-rank tests to compare each individual’s ×3 ×2 successive adjustments with their ×2 ×3 adjustments. The same strategy was used to assess multiplicativity. Note that in all cases, matched-pairs nonparametric tests were employed rather than their counterparts for independent samples, to better account for potential drifts in the adjustments from one trial block to the next.

Since the latter two axioms basically claim that the net result of two different consecutive adjustments (e.g., ×2×3 and ×3×2) should match, which amounts to attempting to show that the null hypothesis holds, a supplementary strategy better suited to assess the likelihood of the null appeared necessary. To that effect, Bayes factors (BF_01_) were computed for each participant’s data using the {BayesFactor} package for R (Morey et al., 2011; Morey & Rouder, 2011; Rouder et al., 2009) in order to determine the likelihood of commutativity (or multiplicativity) to hold (i.e., the null hypothesis; model 0) relative to an axiom violation (the alternative hypothesis; model 1). All Bayes factors were determined with the ttestBF() function for paired observations using wide, and thus relatively uninformed Cauchy prior distributions around a standardized effect size of 0 (width scaled with γ = 1.0; as in Rouder et al., 2009). Thus, as applied to the present analysis, Bayes factors (BF_01_) exceeding 1.0 in principle favor the null hypothesis or the validity of an axiom stating equality. By convention, however, Bayes factors 1/3 < BF_01_ < 3.0 are not considered conclusive evidence for either hypothesis.

### Cross-modal monotonicity

**Fig. 3:**
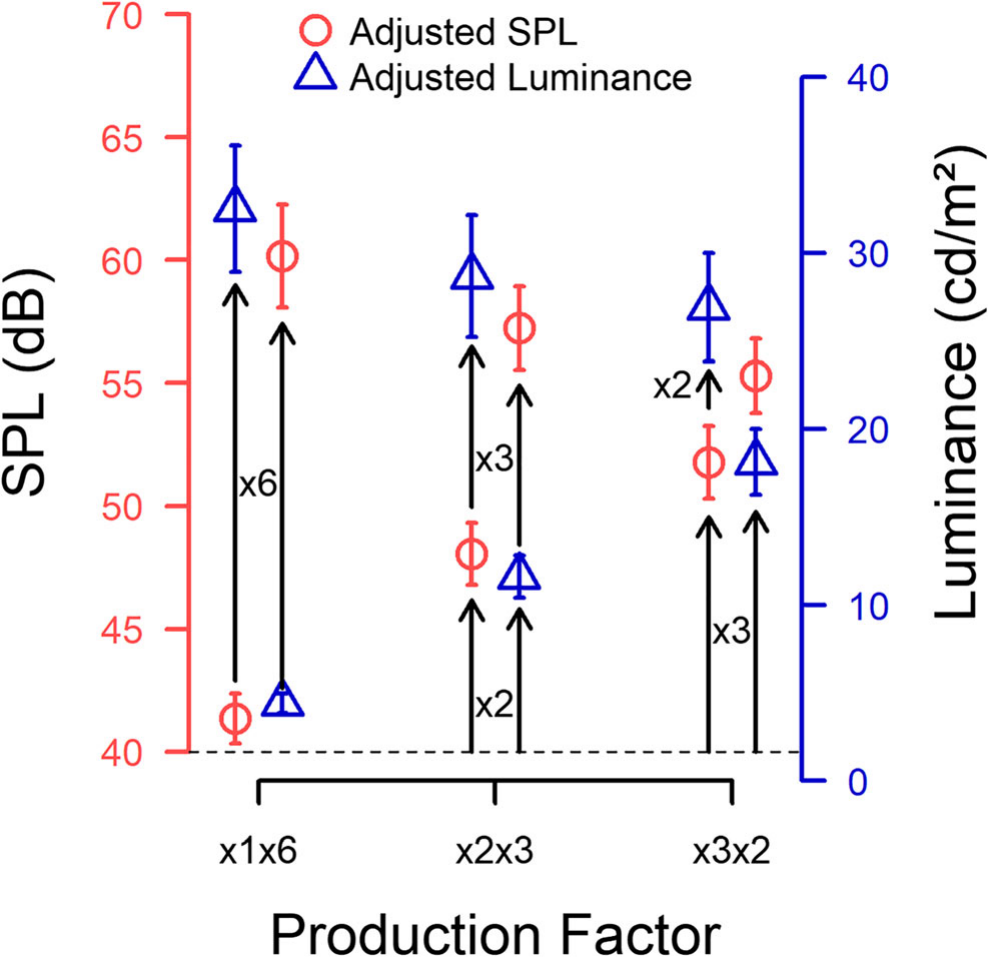
Overall means and standard errors of the basic (lower sets of symbols) and successive (upper sets of symbols) magnitude productions made by all 20 participants in the 12 experimental conditions. The entries on the abscissa denote the different types of instructions given (e.g., ×1×6: making a match on the other dimension first, and then a cross-modal magnitude production resulting in six times the subjective magnitude). Red symbols refer to adjustments of sound pressure level (left ordinate); blue symbols to adjustments of luminance (right ordinate). Naturally, the two types of outcomes cannot be compared directly. The initial magnitude productions originated from a reference sound pressure level of 40 dB(A) and a reference luminance level of 1.66 cd/m^2^ (dashed line). Each data point is based on 280 adjustments

In order to facilitate visualizing the overall empirical outcome of axiom testing, the average adjustments made by all 20 participants in each of the 12 conditions are depicted in Fig. 3, in a slightly different manner than in Fig. 2, i.e., by showing “simple” and “successive” trials as building upon each other. This depiction of overall results shall be used to illustrate the mean descriptive outcome with respect to the validity of a given measurement axiom.

Monotonicity of the adjustments made in the present experiment was evaluated by determining whether the ×1, ×2, and ×3 adjustments in each modality were strictly rank ordered. In Fig. 3, overall monotonicity is evident in the increasing mean stimulus levels adjusted in the two sets of basic trials (lower points in each concatenation of arrows). More critically, monotonicity also held for all mean adjustments made by each individual subject (see Fig. 2). To evaluate it statistically, Friedman analyses of variance by ranks were performed on each participant’s ×1, ×2, and ×3 adjustments, separately for cross-modal productions resulting in a luminance value *x*^*lb*^ (loudness to brightness; the lower blue data points) and for those resulting in a sound pressure level *x*^*bl*^ (brightness to loudness; the lower red data points). All of these analyses were statistically significant, thus justifying pairwise Wilcoxon signed-rank tests on the 14 versus 14 trials per participant to determine which of the three multiplication factors resulted in statistically different adjustments for each subject and modality (see Appendix Table 1). As a result, 102 of the 120 comparisons were statistically significant (*p* < .05). Given that the scattered failures to confirm monotonicity do not seem to be due to specific careless participants, it is concluded that monotonicity essentially holds for all.

### Cross-modal commutativity

Whether cross-modal commutativity (see the left and center columns of Fig. 1) tends to hold, may be inferred from Fig. 3 by comparing the net result of making a ×2×3 (center of figure) versus a ×3×2 adjustment (right part of figure), separately for final adjustments of light (blue triangles) and sound (red circles). Figure 3 suggests that these mean consecutive adjustments converge onto roughly similar stimulus levels. To test whether cross-modal commutativity is valid for each individual subject and for starting out with either modality, Wilcoxon signed rank tests were performed with a significance level of α = .10, since the goal was to support the null hypothesis, and adopting a higher α level will facilitate its rejection.

As may be seen in Appendix Table 2, seven of the 40 tests performed (20 participants starting out from two modalities) showed significant violations of commutativity, typically with the ×2×3 adjustments exceeding the level of the ×3×2 adjustments. Note that with α = .10 per test, some four significant tests in 40 are to be expected by chance alone. Thus, while finding seven significant violations of commutativity in 40 tests slightly exceeds that expectation, note that for only one participant did these occur for both directions of cross-modal production studied (Appendix Table 2).

The outcome of the alternative statistical analysis using Bayesian inference is shown in Appendix Table 2 as well. Consistent with the conclusions drawn from frequentist statistics, almost all Bayes factors suggest that the null hypothesis of commutativity to hold is more likely than an axiom violation (BF_01_ > 1), though often by a small margin. In eight instances, the null hypothesis is found to be more than three times as likely as the alternative hypothesis (BF_01_ > 3; see Appendix Table 2).

### Cross-modal multiplicativity

Multiplicativity requires the successive, cross-modal ×2×3 (or ×3×2) adjustments (left and center columns in Fig. 1) to agree with the successive ×1×6 adjustments (right column in Fig. 1). Figure 3, however, shows the average ×1 ×6 productions (on the left in Fig. 3) to exceed the ×2×3/×3×2 productions by more than one standard error of the mean, casting doubt on the validity of this axiom in the sample studied. To evaluate the statistical significance of individual axiom violations, the average of the ×2 ×3 and the ×3 ×2 productions in a given block of trials was compared with the ×1×6 production from that same block and the pairings thus resulting across the sequence of trial blocks were subjected to Wilcoxon signed-rank tests. All in all, 17 of the 40 tests (20 participants starting out from the two modalities) turned out to reject multiplicativity, see Appendix Table 3.

If we only consider those violations of multiplicativity, for which the prerequisite axiom of commutativity holds (Appendix Table 2), there are still 13 violations in 33 tests. According to the binomial distribution, to obtain such a large number of violations (or a larger one) by chance alone, given α = .10 is highly unlikely, *p* = .00000825.

Bayes factors computed on the raw data of each individual participant in a manner analogous to the previous matched-samples tests favor the null hypothesis (BF_01_ > 1) in the majority of tests, though not as often as in the case of commutativity. In 15 of the 40 tests conducted, however, the null hypothesis of multiplicativity to hold was found to be more than three times as likely as the alternative hypothesis of an axiom violation (BF_01_ > 3; see Appendix Table 3). Note that the ten (of 20) participants for whom both tests result in Bayes factors favoring the null (BF_01_ > 1) are the same ones one would have picked as “satisfying multiplicativity” by visual inspection of Fig. 2.

## Discussion

While Luce et al. (2010) had tested their theory of cross-dimensional magnitude production in situations where a match is performed across physical dimensions, but within the same sensory modality (e.g. using tones of different frequencies), the present study is the first to evaluate psychophysical measurement axioms in a truly cross-modal paradigm, by requiring observers to match – or produce multiples of – brightness sensations to loudness sensations and vice versa. The outcome is discussed (1) by analyzing what it means in terms of Narens’ (1996) axiomatization, (2) by comparing with the available evidence on intra-modal, cross-dimensional magnitude production, and (3) by relating it to the theory of (internal) reference points that potentially complicate matters in the cross-modal situation to a greater extent than in the unimodal case.

### Validity of Narens’ (1996) axioms

Narens’ (1996) axioms of monotonicity, commutativity, and multiplicativity were tested for the cross-modal case, i.e., by mapping sensation multiples from brightness to loudness and vice versa. Note that though Narens’ influential publication bears the title “A Theory of Ratio Magnitude Estimation,” in fact it applies to all varieties of direct magnitude scaling, including cross-modality matching or its extension beyond producing equal sensation strength: cross-modality magnitude production.

As expected, cross-modal monotonicity (Eq. 5) due to numerically ordered instructions (e.g., making the other modality equally (×1), twice (×2), or three times (×3) as intense) held both when mapping sensation multiples from brightness to loudness, and the reverse. Monotonicity, though rarely addressed, has typically been found to hold for single dimensions (visual area: Augustin & Maier, 2008; duration: Birkenbusch et al., 2015; pitch: Kattner & Ellermeier, 2014), and does not imply more than that the observer is operating on an ordinal scale.

Commutativity of cross-modal magnitude productions (Eqs. 3 and 4) was not rejected by conventional null-hypothesis testing in 33 of 40 tests, with violations on the order of what should be expected by chance. Bayesian analysis supports that conclusion in generally finding the null hypothesis (commutativity) to be more likely, though the evidence is somewhat weak and many tests remain inconclusive. That result is consistent with the evaluation of commutativity in many unidimensional situations, where it typically was found to hold in similar proportions based on conventional null-hypothesis testing (Birkenbusch et al., 2015; Ellermeier & Faulhammer, 2000; Kattner & Ellermeier, 2014; Steingrimsson & Luce, 2005; Zimmer, 2005). For the unidimensional case, Narens’ (1996) theory states that commutativity of magnitude productions implies that subjective intensities on the continuum in question may be represented on a ratio scale. Showing commutativity to hold in cross-modal magnitude production goes beyond that: It implies that there is a common underlying ratio scale for both modalities studied (Luce et al., 2010).

The type (e.g., power function) and identity of that scale, however, may only be determined, if multiplicativity (re Eq. 6) holds. Multiplicativity was violated in nearly half of the tests performed on the present data collection, once more in agreement with unidimensional evaluations of this axiom (Birkenbusch et al., 2015; Ellermeier & Faulhammer, 2000; Kattner & Ellermeier, 2014; Steingrimsson & Luce, 2007; Zimmer, 2005). Our present Bayesian analysis, basically concurs, and furthermore suggests to settle the issue of multiplicativity on a case-by-case basis: Note that ten of our 20 participants had Bayes factors favoring the null, i.e., multiplicativity to hold, in both cross-modal directions tested.

According to Narens’ (1996) theory the widespread violation of multiplicativity cautions against interpreting the ‘numerals’ (number words) used by participants in ratio estimation or production to be taken “at face value”: If successive doublings and triplings do not agree with a single sixfold increase, the math just does not work out, or, more specifically, the function linking these numerals to mathematical numbers cannot be the identity function. This problem has been treated in the pertinent literature as one of determining a transformation function (Ellermeier & Faulhammer, 2000; Narens, 1996, Eq. 1; Schneider et al., 1974), a numerical distortion (Birkenbusch & Ellermeier, 2016), or a weighting function (Steingrimsson & Luce, 2007) relating the numerals used in judgments or instructions to their mathematical equivalent. In the present data, that kind of numerical distortion is evident in the fact that for some three quarters of the participants, the ×6 productions systematically exceed the ×2×3 and ×3×2 productions (see Fig. 2), suggesting the function relating numerals to mathematical numbers to be positively accelerated, i.e., the number word “six” to correspond to a “true” mathematical factor > 6. Interestingly, the opposite had been observed in the unidimensional case (Augustin, 2008; Ellermeier & Faulhammer, 2000; Zimmer, 2005) where the ×6 productions typically fell short of the ×2×3/×3×2 productions.

### Potential role of context effects

Attempting to account for the fact that in the present experiment, the ×6 productions not only disagree with the ×2×3/×3×2 adjustments for many participants, but – if anything – systematically exceed them appears to warrant the consideration of potential context effects. Note, however, that if subjects were wary of the high levels associated with a 6× adjustment, the opposite should have occurred. Furthermore, such a “protective” strategy in making adjustments appears unlikely given that the average x6 adjustments in either modality are well below the ceiling level imposed by the apparatus, and also leave plenty of headroom before reaching annoying levels.

Another potential explanation for the direction of the present multiplicativity violations is suggested by studies showing that both (auditory) differential sensitivity and direct perceptual evaluations change depending on the range of stimulus levels expected: Particularly, if rare, unexpected high-level tones are mixed into a series of low-level tones, the latter become less well discriminated (Parker et al., 2002). Even when high-level stimulation is merely implied by verbal instruction, subsequent ratings of mid-level stimuli are strongly attenuated (Parker et al., 2012). By analogy, in the present paradigm, the prospect of being exposed to the high levels required by a ×6 adjustment might lead participants to effectively dampen their psychophysical loudness (or brightness) function, paradoxically requiring even higher levels to produce a ×6 stimulus. As pointed out by one of the reviewers, this kind of plasticity of the psychophysical function with respect to contextual factors is at odds with Narens’ (1996) “cognitive” axiom 5, stating that there is a unique function mapping the objects of perception into sensations.

For both types of “context” explanations elaborated, however, we would have expected these kinds of “protective” mechanisms to be more critical for loudness than for brightness, given that participants are aware of the limited range of luminances that can be produced on a regular computer screen. Contrary to that expectation, the violations of multiplicativity, are essentially parallel no matter whether the final ×6 setting is made by adjusting luminance or sound pressure level (see Fig. 3).

### Ratios versus differences

The fact that for most participants, both cross-modal monotonicity and commutativity held while multiplicativity tended to be violated, might raise the suspicion that participants processed sensory differences rather than ratios when complying with our instructions, since adding numbers is commutative, but not multiplicative (2+3 = 3+2; but 2+3 ≠ 2*3).

The question whether observers can distinguish perceptual ratios from differences at all is a long-standing issue in psychophysics (Schneider et al., 1976; Torgerson, 1961) which – assuming they can *not* – has been labelled “Torgerson’s conjecture.” Interestingly this fundamental issue has also been addressed by the more recent “axiomatic” literature: Narens (1997, 2006) pointed out that Torgerson’s conjecture only appears paradoxical, if the numerals used in instructions to participants (e.g. “produce a sound one-sixth as loud”) are thought to be veridical, i.e. reflecting the corresponding mathematical numbers (1/6). Narens (1997) further suggests that, if subjective ratios and differences turn out to be indistinguishable, they should in fact commute. Evidence for that claim was provided by Ellermeier et al. (2003) who showed that “making a tone p times as loud” exhibited that kind of “generalized commutativity” with the instruction of “adding a certain loudness interval” that was defined by a pair of standard tones successively played on the same trial: Of the 24 stimulus conditions for which commutativity was evaluated in six participants, only six violated commutativity (at *p* < .10). According to Narens’ (2006) theoretical analysis, commutativity of ratio and difference productions implies that both kinds of operations are performed on the same internal ratio scale (Narens, 2006, Theorem 5). Thus, the commutativity of ratio and difference productions in the unidimensional case is further evidence that a literal interpretation of the numerals used by observers is not warranted, and by no means suggests that they judge differences rather than ratios.

### Comparison with the intramodal, cross-dimensional case

While so far we have interpreted our results in terms of Narens’ (1996) theory and by comparing them with the available evidence on testing measurement axioms in unidimensional psychophysics, looking at the two instances in which the “cross-dimensional” theory proposed by Luce et al. (2010) has been put to an empirical test, though on very small samples of participants, seems more pertinent. By defining tones of different frequencies as the two dimensions across which magnitude productions are made (e.g., “Make the high-pitched tone twice as loud as the low-pitched tone!”), Luce et al. found all four participants studied in this condition to satisfy cross-dimensional commutativity (re Eqs. 3 and 4). A very similar result was obtained by Steingrimsson et al. (2012) when the two “dimensions” were luminous squares of different hues (“Make the top square as bright as the bottom square, despite their different color!”). Again all four participants tested for cross-dimensional commutativity from one hue dimension to the other and back (see Eq. 3) generated commutative sequences of ratio productions (Steingrimsson et al., 2012, Fig. 7).

The authors interpret the outcome of both studies as meaning that if *p* and *q* are either multiples or fractions (and not a mix of the two) “individuals rely on a single scale for loudness (brightness) regardless of stimulus frequency (wavelength)” (Luce et al., 2010, p. 6; Steingrimsson et al., 2012, p. 332).

In both studies, however, the net result of a cross-dimensional sequence of productions did not agree with that of a unidimensional such sequence using the same production factors:

7 $$ {x}_{p,q}^{fgf}\sim \kern0.5em {x}_{p,q}^{fff} $$

That inconsistency is interpreted as being due to differing “reference points.”

### The problem of reference points

Different implicit reference points for assessing ratios of subjective intensities had been used as an explanatory construct to account for the often inconsistent results obtained when mixing the production of multiples and fractions to test scaling axioms in the context of Luce’s theory (Birkenbusch & Ellermeier, 2016; Luce et al., 2010). Note that in the case of cross-modal productions, the situation is more complicated: When making a sound three times as loud as a light is bright, for example, the observer may use an (implicit) internal reference on the brightness continuum to determine the brightness magnitude of the reference luminance, and simultaneously a (potentially different) internal reference on the loudness continuum by which to evaluate the adjusted loudness to be three times as intense. Furthermore, these references need not be the same when the match is produced in the other direction, from sound to light. Luce et al. (2010, proposition 4) claim that finding Eq. 7 to hold, implies that these reference points are indeed equal, a condition we did not test in the present experiment. Thus, while the present data support cross-modal commutativity (re Eqs. 3 and 4), and thereby a common underlying scale on both modalities, the question whether observers use the same or discrepant reference points on the two modalities remains open until Eq. 7 is evaluated in cross-modal magnitude production.

For the intramodal, cross-dimensional case, the evidence regarding the equality of reference points is mixed: While Luce et al. (2010) as well as Steingrimsson et al. (2012) found cross-dimensional successive adjustments (left side of Eq. 7) to disagree with unidimensional magnitude productions (right side of Eq. 7), an unpublished study conducted in our laboratory (Schleussner, 2016) investigating the perceived duration of tones of different frequency found successive cross-dimensional productions to agree with unidimensional ones in six of nine participants.

Recently, Heller (2021) in a comprehensive analysis of pertinent theorizing and evidence on cross-modal psychophysics, has pointed out that the reference point issue is even more complicated than envisioned by Luce et al. (2010). Note that in the cross-modal example proposed at the outset of this section, actually four reference points are involved: In Heller’s notation, for a magnitude production from brightness to loudness, we might consider the implicit reference level in the modality of origin, brightness, *ρ*^*b* → *l*^ or the reference level in the target modality, loudness, *ρ*^*l* ← *b*^ with the arrow pointing from the standard to the variable stimulus and the first superscript designating the dimension/modality in which the reference level ρ resides. Likewise, when producing a brightness to be *q* times as intense as a given loudness, the reference levels involved are *ρ*^*l* → *b*^ for the reference level on the modality of origin and *ρ*^*b* ← *l*^ for the reference level on the target modality, brightness. Since the present study verified cross-modal commutativity mapping sensations from brightness (*b*) to loudness (*l*) and back, ${x}_{pq}^{blb}\sim \kern0.5em {x}_{qp}^{blb}$, that implies equal reference points *ρ*^*l* ← *b*^ = *ρ*^*l* → *b*^. Likewise, showing commutativity for the reverse progression across modalities, ${x}_{pq}^{lbl}\sim \kern0.5em {x}_{qp}^{lbl}$ implies *ρ*^*b* ← *l*^ = *ρ*^*b* → *l*^ in Heller’s notation. Thus, while the equality of cross-modal reference points may be determined by the present experiments, their agreement with intra-modal reference points *ρ*^*f* → *f*^ and *ρ* ^*f* ← *f*^ in Eq. 7 still remains to be shown.

Analyzing these situations, Heller (2021) arrived at the conclusion, that in addition to Eq. 7, further tests might be conducted to determine the equivalence of reference points, such as concatenating cross modality matching (establishing equal sensation magnitude, i.e. *p* = 1) with cross-modal magnitude production (producing sensation ratios). These cases should be explored in further empirical work, both intra-modally and by mapping sensations from one sensory modality into another, like in the present study.

### Conclusion

The present experiments show that the majority of observers in a mid-sized sample (N=20) are capable of making truly cross-modal magnitude productions, mapping brightness sensations into loudness and vice versa, that satisfy the commutativity of ratio production factors. That extends the scope beyond the intra-modal, “cross-dimensional” commutativity previously shown (e.g., for luminous patches of different hues) and theoretically analyzed by Luce et al. (2010). Furthermore, a prerequisite for evaluating commutativity, namely, establishing the monotonicity of the effect of ratio production instructions (e.g., ×1, ×2, ×3 as intense), was also demonstrated for the cross-modal case and in the same set of observers. Finally, cross-modal multiplicativity, i.e., finding two successive ratio productions (e.g., ×2 and ×3) to result in the same stimulus level as a cross-modal match (×1) followed by their mathematical product (×6), was not met in almost half of the cases tested. Bayesian analysis, however, by explicitly testing for the confidence to be put in the null hypothesis, qualifies that assertion by showing that in 15 of the 40 tests performed the null hypothesis (multiplicativity) is more than three times more likely than the alternative hypothesis (an axiom violation). In sum, much like in the unimodal case, it may be concluded that observers indeed operate on a common ratio scale when evaluating loudness and brightness, even though the numbers involved may not always be interpreted at “face value.”

## Appendix

**Table 1:** Results of Wilcoxon signed rank tests to evaluate cross-modal monotonicity

	*x*^*lb*^ *(loudness → brightness)*			*x*^*bl*^ *(brightness → loudness)*		
Subject	*p* (1-2) ^a^	*p* (2-3)	*p* (1-3)	*p* (1-2)	*p* (2-3)	*p* (1-3)
HOED	.002	.009	.002	.002	.004	.002
MINI23	**.158**	**.089**	.013	.002	.004	.002
KLCH06	.002	.002	.002	.002	**.320**	.002
VOSO31	.003	.017	.001	.002	.009	.002
HEKA29	.020	.020	.006	.003	.022	.003
DIAN07	.002	.006	.002	.002	.004	.002
ZESI15	.004	**.058**	.005	.002	**.156**	.002
SESV24	.001	.001	.001	.010	.018	.005
IGMA08	.005	.011	.003	.002	.044	.002
HAKA24	.003	**.079**	.003	.008	.014	.004
HEAN19	**.173**	**.173**	.021	.007	.025	.005
FRBR05	**.258**	**.178**	.032	.028	.021	.007
ECGE12	**.109**	.009	.009	.012	.007	.005
KAGA10	.009	.003	.002	.003	.014	.003
RUMA10	.011	**.084**	.003	.010	**.107**	.003
SEIR22	.018	**.078**	.016	.016	.006	.006
JEGR05	.002	.044	.000	.002	**.054**	.002
STRE25	.029	.038	.009	.017	**.139**	.004
FRAL10	.016	**.330**	.020	**.084**	.022	.012
THMA12	.002	.017	.002	.002	.025	.002

*Note.* Bold type indicates *p*>.05.

^a^The ratio production factors compared are given in parentheses with the *p* values. Superscripts *lb* (left half of the table) refer to cross-modal magnitude productions from loudness *(l*) to brightness (*b*), superscripts *bl* to the reverse operation (right half of the table)

**Table 2:** Wilcoxon signed-ranks tests indicating violations of cross-modal commutativity and corresponding Bayes factors in favor of the null hypothesis of the axiom to hold (BF_01_)

	Wilcoxon test		Bayes factor	Wilcoxon test		Bayes factor
	*X*^*blb*^ *(b → l → b)*			*X*^*lbl*^ *(l → b → l)*		
Subject	*z(V)*	*p*	*BF*_*01*_	*z(V)*	*p*	*BF*_*01*_
HOED	-1.88	**.060**	0.720	-0.85	.409	2.776
MINI23	0.69	.490	2.762	-1.80	**.094**	0.978
KLCH06	-1.57	.116	2.182	0	>.999	4.977
VOSO31	0.06	.950	2.831	-1.14	.256	2.540
HEKA29	0.85	.396	2.236	-1.37	.166	1.397
DIAN07	-1.76	**.079**	2.015	-2.07	**.039**	1.114
ZESI15	-0.38	.715	2.562	-0.91	.362	3.420
SESV24	-1.26	.208	1.682	-1.58	.115	1.129
IGMA08	-2.32	**.020**	0.574	0	>.999	4.575
HAKA24	-0.66	.509	2.384	-0.69	.489	3.829
HEAN19	-1.00	.326	2.007	-0.77	.441	3.032
FRBR05	-2.07	**.035**	0.581	-0.50	.615	3.511
ECGE12	0.13	.900	2.723	-1.30	.195	1.757
KAGA10	-0.63	.530	2.802	1.57	.116	1.905
RUMA10	-1.13	.258	2.680	-1.47	.141	1.563
SEIR22	1.61	.108	1.028	-1.30	.195	2.033
JEGR05	-0.31	.753	2.483	-1.07	.283	3.252
STRE25	-2.98	**.003**	0.161	-0.63	.529	4.332
FRAL10	0	>.999	2.798	-1.51	.131	1.254
THMA12	1.57	.119	0.929	-1.04	.298	2.151

*Note. z*-scores for the Wilcoxon test statistic *V* and corresponding *p*-values are reported. All *p* < 0.1 shown in bold type. Bayes factors indicate how likely the null hypothesis of the axiom to hold is, compared to the alternative hypothesis of a violation of commutativity

**Table 3:** Wilcoxon signed-ranks tests for violations of cross-modal multiplicativity and corresponding Bayes factors in favor of the null hypothesis of the axiom to hold (BF_01_)

	Wilcoxon test		Bayes factor	Wilcoxon test		Bayes factor
	*X*^*blb*^			*X*^*lbl*^		
Subject	*z(V)*	*P*	*BF*_*01*_	*z(V)*	*p*	*BF*_*01*_
HOED	-0.42	.673	2.638	-1.94	**.052**	1.094
MINI23	0.63	.529	4.071	-3.26	**.001**	0.006
KLCH06	-0.63	.542	4.335	-0.60	.550	3.491
VOSO31	-1.51	.135	1.574	-2.63	**.009**	0.036
HEKA29	-2.07	**.035**	0.782	-1.86	**.064**	2.042
DIAN07	-2.51	**.009**	0.246	-3.26	**.001**	0.0002
ZESI15	-1.07	.296	3.022	-2.51	**.009**	0.752
SESV24	-1.26	.209	2.157	1.26	.209	1.380
IGMA08	-1.01	.315	4.250	-1.04	.298	3.348
HAKA24	-1.88	**.058**	0.704	-2.45	**.014**	0.291
HEAN19	-0.57	.583	3.734	-2.01	**.045**	1.776
FRBR05	-1.57	.116	1.260	-2.83	**.002**	0.514
ECGE12	-1.30	.209	1.585	-2.56	**.011**	0.357
KAGA10	-0.13	.903	4.987	0.94	.345	4.980
RUMA10	-1.26	.217	3.448	0.28	.777	2.510
SEIR22	-0.88	.391	3.768	-1.70	**.090**	2.196
JEGR05	-0.31	.753	4.963	-1.97	**.049**	2.443
STRE25	-1.95	**.049**	0.824	-0.67	.506	4.059
FRAL10	-2.20	**.028**	0.568	-0.91	.362	4.584
THMA12	0.66	.510	2.489	-1.42	.157	4.899

*Note. z*-scores for the Wilcoxon test statistic *V* and corresponding *p*-values are reported. All *p* < 0.1 shown in bold type. Bayes factors indicate how much more likely the null hypothesis of the axiom to hold is, compared to the alternative hypothesis of a violation of multiplicativity
